# Decreased ADAMTS 13 Activity is Associated With Disease Severity and Outcome in Pediatric Severe Sepsis

**DOI:** 10.1097/MD.0000000000003374

**Published:** 2016-04-22

**Authors:** Jainn-Jim Lin, Oi-Wa Chan, Hsiang-Ju Hsiao, Yu Wang, Shao-Hsuan Hsia, Cheng-Hsun Chiu

**Affiliations:** From the Division of Pediatric Critical Care and Pediatric Sepsis Study Group (J-JL, O-WC, S-HH), Department of Pediatrics, Chang Gung Memorial Hospital, Keelung; College of Medicine (H-JH), Chang Gung University, Taoyuan; Department of Pediatrics (YW), Chang Gung Memorial Hospital, Chiayi; Graduate Institute of Clinical Medical Sciences (J-JL), College of Medicine, Chang Gung University; Division of Pediatric Infection (C-HC, J-JL); and Molecular Infectious Disease Research Center (C-HC, J-JL), Chang Gung Children's Hospital and Chang Gung Memorial Hospital, Chang Gung University College of Medicine, Taoyuan, Taiwan.

## Abstract

Decreased ADAMTS 13 activity has been reported in severe sepsis and in sepsis-induced disseminated intravascular coagulation. This study aimed to investigate the role of ADAMTS 13 in different pediatric sepsis syndromes and evaluate its relationship with disease severity and outcome.

We prospectively collected cases of sepsis treated in a pediatric intensive care unit, between July 2012 and June 2014 in Chang Gung Children's Hospital in Taoyuan, Taiwan. Clinical characteristics and ADAMTS-13 activity were analyzed.

All sepsis syndromes had decreased ADAMTS 13 activity on days 1 and 3 of admission compared to healthy controls. Patients with septic shock had significantly decreased ADAMTS 13 activity on days 1 and 3 compared to those with sepsis and severe sepsis. There was a significant negative correlation between ADAMTS 13 activity on day 1 and day 1 PRISM-II, PELOD, P-MOD, and DIC scores. Patients with mortality had significantly decreased ADAMTS 13 activity on day 1 than survivors, but not on day 3.

Different pediatric sepsis syndromes have varying degrees of decreased ADAMTS 13 activity. ADAMTS 13 activity is strongly negatively correlated with disease severity of pediatric sepsis syndrome, whereas decreased ADAMTS 13 activity on day 1 is associated with increased risk of mortality.

## INTRODUCTION

Sepsis is a clinical syndrome characterized by systemic inflammatory response secondary to infection. It is one of the leading causes of admissions to the pediatric intensive care unit.^1^ Severe sepsis is frequently complicated by septic shock and multiorgan dysfunction syndrome. Despite increased knowledge on sepsis and decreased mortality by septic shock and multiorgan failure over the last decade, the overall mortality rate remains unacceptably high.^2^

In the pathophysiology of sepsis, endothelial dysfunction plays an important role. Under normal conditions, endothelial cells inhibit coagulation, prevent platelet aggregation, and regulate vascular tone and permeability. During sepsis, endothelial activation induces a pro-coagulant state that is associated with widespread microvascular thrombosis.^3,4^ The von Willebrand factor (vWF) is involved in this process by mediating platelet adhesion and aggregation at sites of vascular injury. It is released from the stimulated endothelium to form the hyperactive and ultralarge von Willebrand factor (UL-vWF).^3–5^

However, vWF functions differently depending on its multimeric size and adhesive properties, which are regulated by a disintegrin and metalloprotease with a thrombospondin type 1 motif, member 13 (ADAMTS 13). ADAMTS 13 is a protease that cleaves the UL-vWF and converts it to smaller, less active vWF multimers in circulation.^5^ Thus, the decrease in ADAMTS 13 activity results in the persistence of UL-vWF and the formation of microvascular thrombi, resulting in microvascular ischemia and organ failure.

The clinical relevance and prognostic association of ADAMTS 13 activity during different sepsis syndrome remain uncertain. Recently, some studies have shown decreased activity or antigen levels of ADAMTS 13 during severe sepsis associated with disease severity and outcome.^6–14^ But ADAMTS 13 activity in different sepsis syndromes has not been widely investigated in children except for pediatric meningococcus sepsis.^10^ This study aimed to investigate the role of ADAMTS 13 activity in different pediatric sepsis syndromes and evaluate the relationship with disease severity and outcome. To overcome the diversity of underlying diseases, this study focus on previously healthy children who presented with sepsis.

## PATIENTS AND METHODS

### Design, Patients, and ICU Setting

This prospective 24 months study from July 2012 to June 2014 was conducted in the 29-bed pediatric intensive care unit (PICU) of Chang Gung Children's hospital. Previous healthy patients aged <18 years old admitted to the PICU and diagnosed as having sepsis within 24 hours of admission were recruited and divided to 3 groups: sepsis, severe sepsis, and septic shock, which were defined according to the guidelines of the society of Critical Care Medicine Consensus Conference Committee.^15^ Patients with known underlying diseases such as congenital heart disease, chronic lung disease, encephalopathy, rheumatologic disease, thrombotic or bleeding disorder, and liver disease, or those who received blood transfusion within a week of sample collection were excluded from the study. Controls were recruited from healthy children age 5 to 8 years in the outpatient clinic for tic disorders.

The Ethical Review Committee of Chang Gung Memorial Hospital Approved the study (IRB Nos. 100-2984A3, 103-3076C, 103-4188C, and 104-3052C). All the participants or their parents provided written informed consent.

The information collected included age, sex, hematologic and biochemical tests, site and organism of infection, mechanical ventilation requirement, vasopressor use, disease severity score on day 1 of PICU admission using the Pediatric Risk of Mortality (PRISM) II, Pediatric Logistic Organ Dysfunction (PELOD), and Pediatric-Multiple Organ Dysfunction (P-MOD) scores, and disseminated intravascular coagulation (DIC) based on the International Society for Thrombosis and Hemostasis score.

### Sample Collection

Venous blood (3 mL) was taken from every patient on days 1 and 3 of admission. Day 3 samples were collected for survivors by day 3. Platelet poor plasma was obtained after centrifugation. The plasma samples were labeled and kept at −80°C until analysis.

### ADAMTS 13, vWF, and Cytokine Profile Level Analyses

ADAMTS 13 activity was assessed by a commercially available chromogenic enzyme-linked immunosorbent assay (ELISA) (TECHNOZYM^®^ ADAMTS-13 activity, Technoclone, Vienna, Austria), according to the manufacturer's instructions. ADAMTS-13 activity <40% was defined as deficiency by the manufacturer. Plasma vWF levels were measured using IMUBIND^®^ vWF ELISA kits (Sekisui Diagnostics, Stamford, CT). Cytokine profile analysis, including interleukin-4, interleukin-6, interferon-γ, and tumor necrosis factor-α, were measured simultaneously using a Human Th1/Th2 11plex FlowCytomix kit (eBioscience, Bender MedSystems, Austria) according to the manufacturer's instructions.

### Statistical Analysis

Statistical analysis was performed using the SPSS statistical software, version 12.0 (SPSS, Inc, Chicago, IL). Descriptive data were presented as mean ± standard deviation (SD) or percentage. Differences in ADAMTS 13 activity and vWF level between the different sepsis syndromes and healthy controls were analyzed. The Mann–Whitney *U* test and Kruskal–Wallis test were used for continuous variables, whereas the chi-square or Fisher's exact test was used for categorical variables. Spearman's rank correlation coefficient was used to evaluate the correlation between ADATMS 13 activity and severity score and other laboratory parameters. The areas under the receiver operating characteristic curves (AUC) and cut-off points for day 1 serum ADAMTS 13 and vWF level to predict the development of mortality were calculated. Statistical significance was set at *P* < 0.05. All statistical tests were 2-tailed.

## RESULTS

### Demographic Characteristics

**FIGURE 1:**
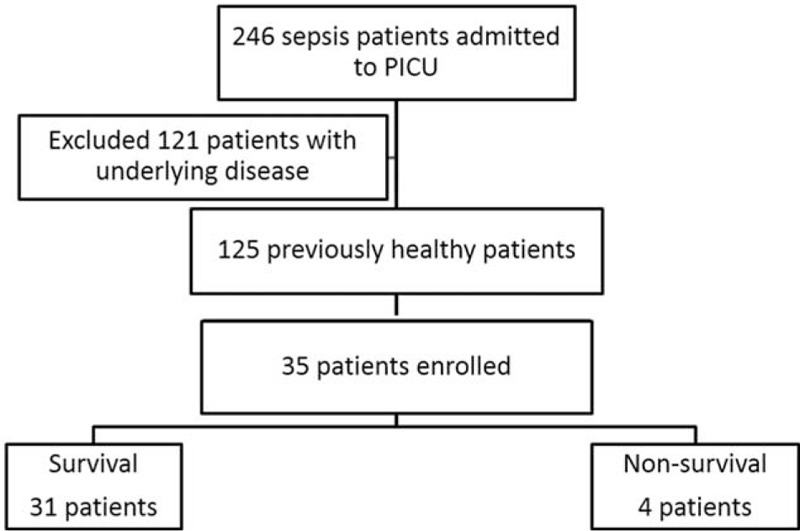
Flowchart of the study.

The 35 patients with sepsis included 20 (57.1%) men and 15 (42.9%) women and had a median age of 5.51 years (range, 2 month to 16 years 10 months) (Figure 1). Nine (25.7%) had sepsis, 12 (34.3%) had severe sepsis, and 14 (40%) had septic shock. Microbiologically defined infection was present in 19 (54.3%) patients, including *Streptococcus pneumoniae* in 16 (45.7%), *Escherichia coli* in 1 (2.9%), *Samonella group D* in 1 (2.9%), and *Mycoplasma pneumoniae* in 1 (2.9%). The most frequent infection sites were the respiratory system (n = 15, 42.9%), central nervous system (n = 5, 14.3%) patients, and the heart (n = 4, 11.4%). Besides, in our study, only 1 patient with invasive *S pneumoniae* infection developed atypical hemolytic uremic syndrome later on during the hospital stay. And the level of ADAMTS 13 activity is relative low (45.57% and 49.86%) on days 1 and 3.

Twelve (34.3%) patients had platelet count <100,000/μL on admission, including 7 (58.3%) in the septic shock group and 5 (41.7%) in the severe sepsis group. The platelet count on admission in the septic shock group (123.8 ± 98.3 × 103/μL) was lower than that in the sepsis and severe sepsis groups (258.3 ± 88.4 × 103/μL and 214.7 ± 196.0 × 103/μL, respectively). However, this did not reach statistical significance (*P* = 0.07). ADAMTS 13 activity in patients with platelet count <100,000/μL was significantly lower than that in patients with higher platelet counts (55.35 ± 2.07% vs 65.72 ± 20.63%), but also not statistically significant (*P* = 0.17).

Eighteen (51.4%) patients needed mechanical ventilator support and 14(40%) received inotropic agents. There were significant differences in the disease severity among different sepsis syndrome as assessed by the cytokine profile, PRISM-II, PELOD, P-MODS, and DIC scores. The duration of ICU stay and hospitalization were 7.11 ± 3.76 and 17.11 ± 12.07 days, respectively, in the sepsis group, 10.36 ± 6.09 and 20.45 ± 11.17 days, respectively, in the severe sepsis group, and 11.79 ± 9.90 and 20.71 ± 14.19 days, respectively, in the septic shock group. However, the difference was not statistically significant. The demographic data of different pediatric sepsis syndromes were listed in Table 1.

**TABLE 1:**
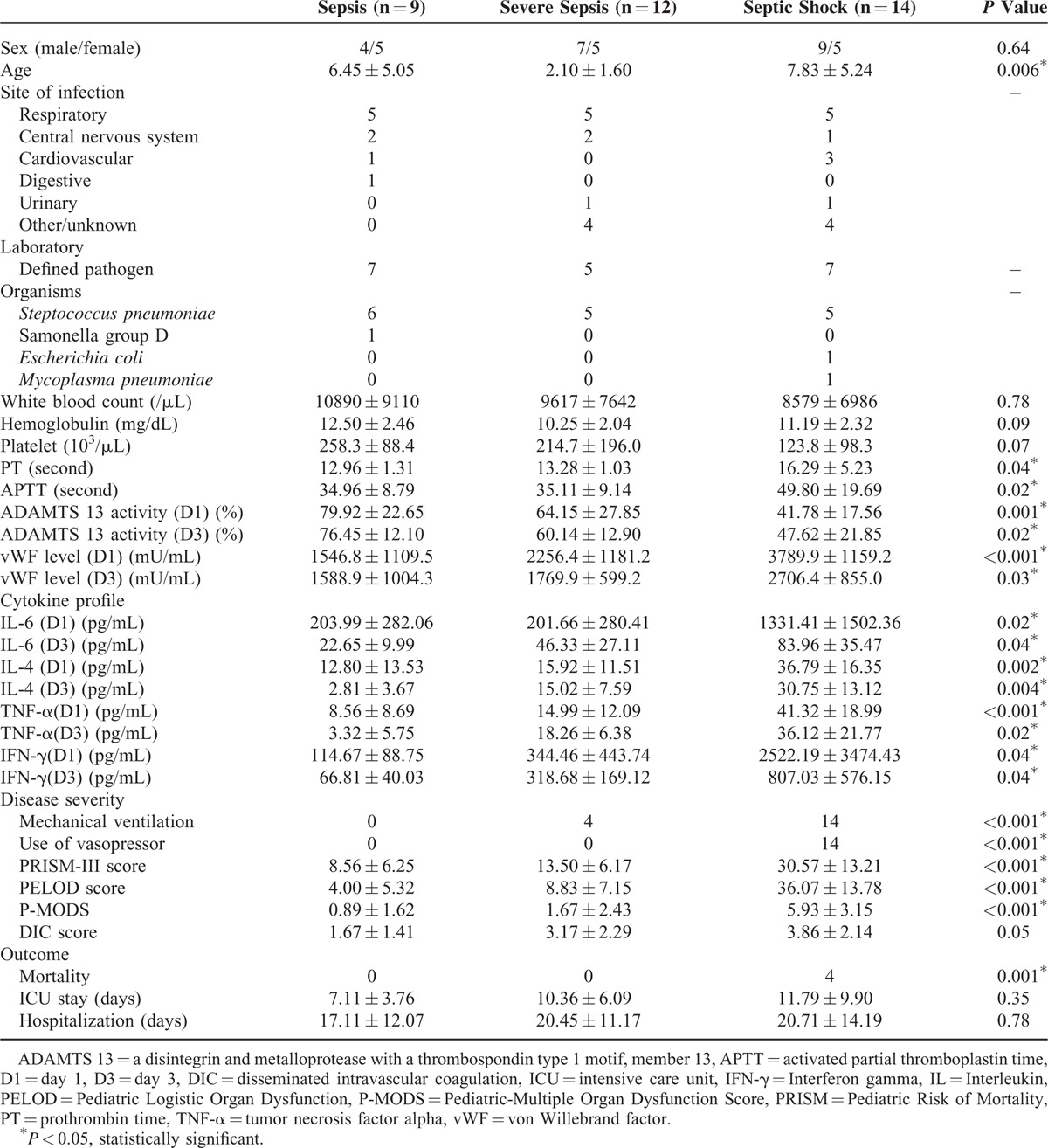
Demographic Data and Pathogens of Patients in Different Sepsis Syndromes (n = 35)

### ADAMTS 13 Activity and Disease Severity

**FIGURE 2:**
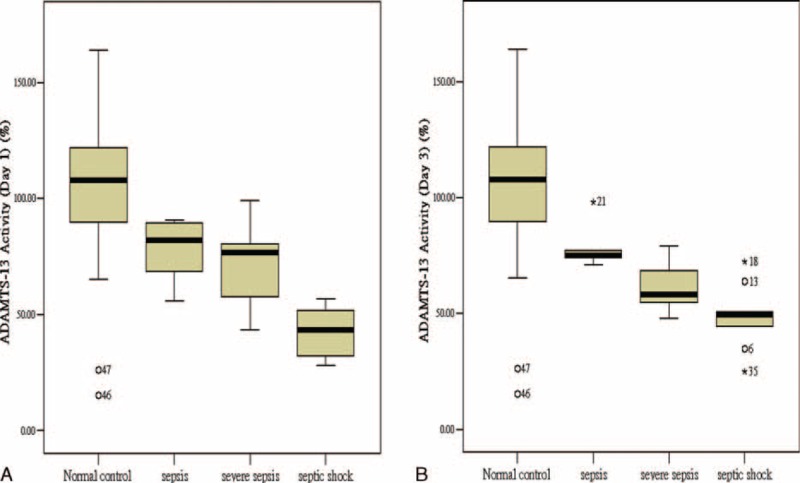
The level of ADAMTS 13 activity in different sepsis syndromes compared to healthy controls. All different sepsis syndromes had decreased ADAMTS 13 activity on (A) day 1 and (B) day 3 of admission compared to healthy controls (all group *P* < 0.001). The ADAMTS-13 activity on days 1 and 3 was significantly lower in the septic shock group than in the sepsis and severe sepsis group (*P* = 0.001 and 0.026, respectively). ADAMTS 13 = a disintegrin and metalloprotease with a thrombospondin type 1 motif, member 13.

All different sepsis syndromes had decreased ADAMTS 13 activity on days 1 and 3 of admission compared to the healthy control group (105.63 ± 18.30, all group *P* < 0.001) (Figure 2A and B). Four (16%) patients had ADAMTS 13 deficiency on admission (activity <40%). The ADAMTS 13 activity on days 1 and 3 was significantly lower in the septic shock group than in the sepsis and severe sepsis groups (*P* = 0.001 and 0.02, respectively). The level between ADAMTS 13 activity and vWF as a measure for the relation between the protease and its substrate was strongly negatively correlated with different sepsis syndromes (Table 1) and compared to controls (698.71 ± 392.04 mU/mL) (*P* < 0.001, no shown). There was also a strong negative correlation between ADAMTS 13 activity on day 1 and cytokine profile, baseline PRISM-II, PELOD, P-MODS, and DIC scores. The correlation of ADAMTS 13 activity and disease severity was summarized in Table 2.

**TABLE 2:**
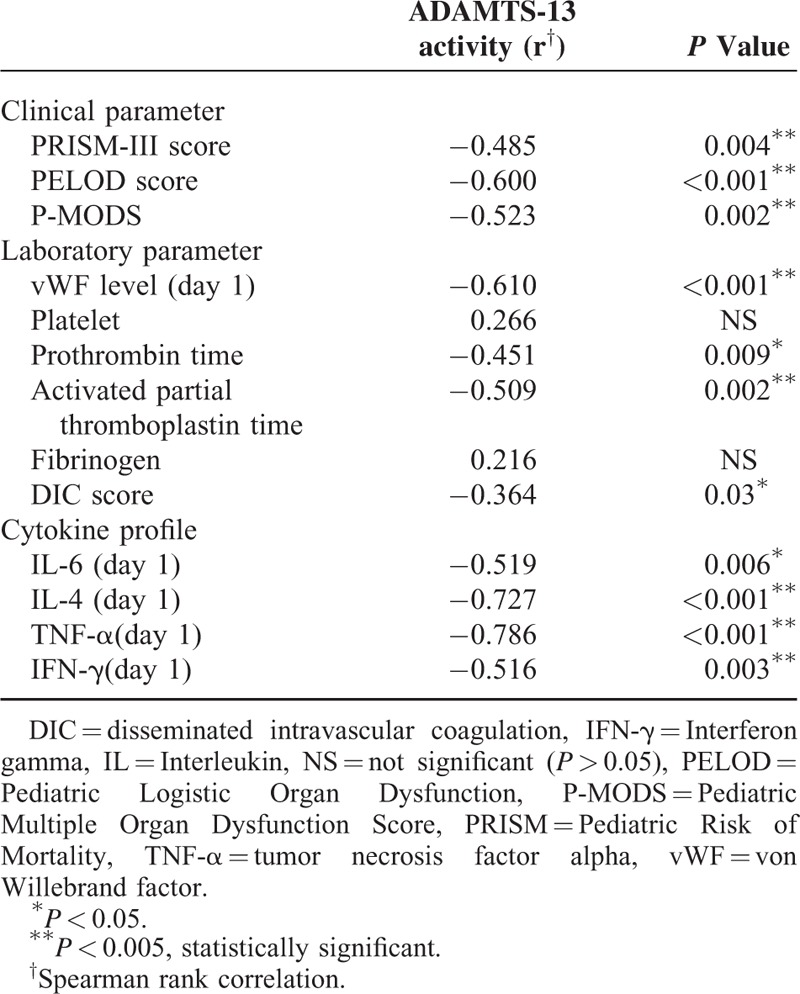
Correlation Between Day 1 ADAMTS-13 Activity and Disease Severity and Laboratory Parameters

### ADAMTS13 Activity and Mortality

In-hospital mortality was seen in 4 (11.4%) patients, all of whom were in the septic shock group. Mortality was significantly higher in the septic shock group compared to the sepsis and severe sepsis groups (*P* = 0.001). The ADAMTS 13 activity on day 1 of admission in the nonsurvival group was 44.07 ± 10.99%, which was significantly lower in the survival group at 64.42 ± 20.66% (*P* = 0.02). However, the ADAMTS 13 activity on day 3 of admission in the nonsurvival group did not differ from that of the survival group (53.56 ± 26.59 vs 60.94 ± 16.80%, *P* = 0.57) (Table 3).

**TABLE 3:**
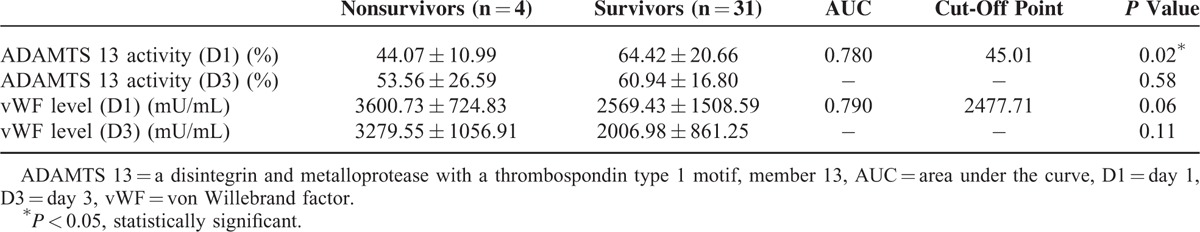
The ADAMTS 13 Activity, vWF Level, and Mortality

Areas under the ROC curves (AUC) and cut-off points for serum ADAMTS 13 and vWF levels on day 1 of sepsis in predicting mortality are shown in Table 3. The areas under the ROC curves and cut-off points showed that day 1 serum ADAMTS 13 levels had good discriminative power for the development of mortality (AUC = 0.780, cut-off point 45.01%).

## DISCUSSION

This study analyzed ADAMTS 13 activity in different pediatric sepsis syndromes, including sepsis, severe sepsis, and septic shock, and assessed the ADAMTS 13 activity in relation to the disease severity of different sepsis syndromes and outcomes. There was decreased ADAMTS 13 activity in all pediatric sepsis syndromes compared to healthy controls. Four (16%) patients had ADAMTS 13 deficiency on admission (<40%). ADAMTS 13 activity was strongly negatively correlated to disease severity of pediatric sepsis. Decreased ADAMTS 13 activity on day 1 of admission was also associated with increased risk of mortality.

The incidence of severe ADAMTS 13 deficiency in pediatric sepsis is unknown. Severe ADAMTS 13 deficiency is seen in thrombotic thrombocytopenic purpura, although a mild-to-moderate decrease in ADAMTS 13 activity or antigen has been described in adult and pediatric patients with severe sepsis.^6–14^ Because elevated levels of vWF are observed in sepsis, normal or mildly decreased ADAMTS 13 activity may not be sufficient enough to control vWF multimer size.^9^ Ono et al reported decreased ADAMTS 13 activity (<5%) in 15.6% of patients with sepsis induced by disseminated intravascular coagulation.^6^ Peigne et al focused on ADAMTS 13 in septic shock and found that 50% had ADAMTS 13 deficiency.^13^ However, Kremer et al reported no severe or borderline ADAMTS 13 deficiency in their patients with severe sepsis and septic shock.^9^

In pediatric studies with severe sepsis, the incidence of ADAMTS 13 deficiency is 31% to 65%.^7,11^ This study focused on previous healthy pediatric patients with different sepsis syndromes and demonstrated a decrease in ADAMTS 13 activity in all pediatric sepsis syndromes compared to healthy controls. Only 4 children (16%) had ADAMTS 13 deficiency (activity <40%).

The role of ADAMTS 13 in disease severity and outcome of sepsis is uncertain in adults. Kremer et al reported ADAMTS 13 in 40 adult patients with sepsis, but did not find any correlation between disease severity scores (logistic organ dysfunction and simplified acute physiology scores) and outcomes with ADAMTS 13.^9^ In contrast, Aibar et al reported ADAMTS 13 in 121 adult patients with septic syndromes and found a significant negative correlation between ADAMTS 13 level and changes in sepsis-related organ failure assessment (SOFA) and APACHE II scores on admission.^14^ Fukushima et al conducted a study of ratio of von Willebrand factor pro-peptide (vWF-pp) to ADAMTS 13 in patients with severe sepsis or septic shock and found that the vWF-pp/ADAMTS 13 ratio was associated with disease severity and could help identify patients at risk of multiple organ dysfunction.^12^ Kremer et al reported that among patients with severe sepsis, those above the median of ADAMTS 13 activity had better survival compared to those below the median.^9^

The role ADAMTS 13 in the disease severity and outcome of pediatric sepsis syndromes is not widely investigated. Bongers et al reported 58 children with severe meningococcal sepsis and found that ADAMTS 13 correlated with disease severity (Pediatric Risk of Mortality score) and survival outcome.^10^ Karim et al did not find any association between ADAMTS 13 deficiency and in-hospital mortality in 90 pediatric patients with severe sepsis.^11^

In this study, there was decreased ADAMTS 13 activity in all the patients with different sepsis syndromes compared to the healthy control group. ADAMTS 13 activity strongly negatively correlated with disease severity. Lower ADAMTS 13 activity on day 1 of admission was also associated with increased risk of mortality. Thus, ADAMTS 13 activity may be used as a diagnostic and prognosis marker in different pediatric sepsis syndromes.

ADAMTS 13 deficiency has also been reported to play an important role in sepsis-associated thrombocytopenia, which is correlated with poor prognosis.^7^ Nguyen et al evaluated 21 pediatric patients with severe sepsis and concluded that 71.4% of sepsis-associated thrombocytopenia was found in patients with severe ADAMTS 13 deficiency.^7^ They also focused on children with thrombocytopenia-associated multiple organ failure and found that mortality was associated with reduced ADAMTS 13 activity.^16^

Karim et al also found that 75.6% of sepsis-associated thrombocytopenia was in the ADAMTS 13 deficient group.^11^ However, in this study, ADAMTS 13 activity in patients with platelet count <100,000/μL was significantly lower than that in patients with higher platelet counts (55.35 ± 2.07% vs 65.72 ± 20.63%), but not statistically significant (*P* = 0.17). Further studies are warranted.

Decreased ADAMTS 13 activity has been reported in *S pneumoniae* associated hemolytic uremic syndrome. Pelras et al report a 2-year-old girl with severe ADAMTS 13 deficiency in pneumococcal-associated hemolytic uremic syndrome.^17^ In our study, only 1 patient with *S pneumoniae* infection developed atypical hemolytic uremic syndrome. And the level of ADAMTS 13 activity is relative low on days 1 and 3. Further large study is needed to confirm the role of ADAMTS 13 activity in the *S pneumoniae* associated hemolytic uremic syndrome.

### Study Limitations

This study has some limitations. First, this is a small prospective study. There were only 35 study patients and ADAMTS 13 was only assayed on days 1 and 3 after admission. Serial monitoring was not done so the patients’ evolution in the PICU could not be assessed. Second, the definition of severe deficiency was different, depending on the method used such that different study populations had different incidences. Thus, comparing the incidences of ADAMTS 13 in different pediatric sepsis studies is difficult. More focused studies are needed. Third, most of the microbiologically-confirmed infection in our study was resulted from *Streptococcus pneumoniae*. And respiratory infection/pneumonia is a primary cause of admission. Whether the decrease of ADAMTS 13 activity is a biological event specific to pneumococcal and/or respiratory infections, further studies are warranted. Lastly, the 15 controls are from outpatient clinic for tic disorders. Whether the ADAMTS 13 activity is normal in this population is unknown. Age- and sex-matched healthy controls may be more appropriate.

## CONCLUSIONS

All pediatric sepsis syndromes have decreased ADAMTS 13 activity compared to healthy controls and very few (only 16%) exhibit ADAMTS 13deficiency (activity <40%). ADAMTS 13 activity is strongly negatively correlated with the severity of pediatric sepsis. Lower ADAMTS 13 activity on day 1 of admission is associated with increased risk of mortality. Therefore, analysis of ADAMTS 13 activity in pediatric sepsis may offer help in evaluating the status and outcome of patients.
